# Similarity Evaluation of Different Origins and Species of Dendrobiums by GC-MS and FTIR Analysis of Polysaccharides

**DOI:** 10.1155/2015/713410

**Published:** 2015-10-11

**Authors:** Nai-Dong Chen, Nai-Fu Chen, Jun Li, Cai-Yun Cao, Jin-Mei Wang, He-Ping Huang

**Affiliations:** ^1^College of Biotechnology and Pharmaceutical Engineering, West Anhui University, Lu'an 237012, China; ^2^West Anhui Biotechnology Research Center of Natural Medicine and Traditional Chinese Medicine, West Anhui University, Lu'an 237012, China; ^3^School of Pharmacy, Anhui Medical University, Hefei 230032, China; ^4^School of Pharmacy, Anhui University of Chinese Medicine, Hefei 230012, China

## Abstract

GC-MS method combined with FTIR techniques by the analysis of polysaccharide was applied to evaluate the similarity between wild (W) and tissue-cultured (TC)* Dendrobium huoshanense *(DHS),* Dendrobium officinale *(DO), and* Dendrobium moniliforme* (DM) as well as 3 wild* Dendrobium* spp.:* Dendrobium henanense *(DHN),* Dendrobium loddigesii *(DL), and* Dendrobium crepidatum* (DC). Eight monosaccharides involving xylose, arabinose, rhamnose, glucose, mannose, fructose, galactose, and galacturonic acid were identified in the polysaccharide from each* Dendrobium* sample while the contents of the monosugars varied remarkably across origins and species. Further similarity evaluation based on GC-MS data showed that the *r*_cor_ values of different origins of DHS, DO, and DM were 0.831, 0.865, and 0.884, respectively, while the *r*_cor_ values ranged from 0.475 to 0.837 across species. FTIR files of the polysaccharides revealed that the similarity coefficients between W and TC-DHS, DO, and DM were 88.7%, 86.8%, and 88.5%, respectively, in contrast to the similarity coefficients varying from 57.4% to 82.6% across species. These results suggested that the structures of polysaccharides between different origins of the investigated* Dendrobiums* might be higher than what we had supposed.

## 1. Introduction

*Dendrobium *is the second largest genus in the family of Orchidaceae [1, 2]. More than 70 species of* Dendrobium *are found in China [3]. The stems of some dendrobiums, including* D. huoshanense*,* D. officinale*,* D. moniliforme*,* D. loddigesii*, and* D. crepidatum*, are abundant in active compounds, especially in polysaccharides [4], and have been used as herbal medicines and nutraceutical products in China due to the potential benefiting effect to the stomach, moistening the lung, and immune-stimulating, antitumor, and antimutagenic activities [5–7]. Therefore, the demand for medicinal dendrobiums had constantly been increasing and resulted in overexploitation and depletion of some wild plant resources such as* D. huoshanense*,* D. officinale*, and* D. moniliforme* and left them critically endangered. In the traditional product region, tissue-cultured dendrobiums have already become the major resource of pharmaceutical dendrobiums. As plant amedica, it is extremely vital for tissue-cultured medicinal plants to obtain the ability to produce similar chemical components to their wild correspondences. Establishing a fast quality-identification method to evaluate the similarity of wild and tissue-cultured dendrobiums is a critical step for assurance of quality and safety in the TCM industry.

So far, the published phytochemical experiments have demonstrated that the bioactivities of polysaccharide were closely related to the level of monosaccharide compositions and the poly/monosaccharide ratio [8]. The analysis of monosaccharide compositions of polysaccharide is the most important step for further discovery of its physicochemical properties, structure, and structure-bioactivity relationship and thus is an essential parameter for the quality evaluation of polysaccharide-based drugs and food ingredients with specific health enhancing functions [3, 9]. Pharmacological studies revealed that, as one of the major bioactive components of* Dendrobium* plants,* Dendrobium *polysaccharide showed many beneficial effects on human physiology such as the immune-stimulatory properties [10, 11], immunoenhancing properties [9], and antitumor properties [12–14]. Therefore, it is essential to have information about whether the monosaccharide compositions and contents are similar among different origins and species of dendrobiums.

In this study, as one of our serial efforts on similarity evaluations of tissue-cultured dendrobiums and their wild correspondences, GC-MS and FTIR were applied to analyze the polysaccharides among different origins of* D. huoshanense*,* D. officinale*, and* D. moniliforme *as well as another 3 wild* Dendrobium* species. The fingerprints, based on GC-MS method combined with FTIR analysis, offered comprehensive information of the polysaccharides from Chinese herbals and would be useful for the quality assessment of medicinal dendrobiums of different origins and species and provide new insights into the utilization and conservation of rare and endangered medicinal plants by tissue-culture techniques.

## 2. Experimental

### 2.1. Plant Materials

The current season's vegetative stems of tissue-cultured and wild* D. huoshanense*,* D. officinale*, and* D. moniliforme* as well as wild* D. henanense* (Table 1) were collected in October 2013 from Huoshan County, Anhui Province, China. Wild* D. loddigesii* and* D. crepidatum* were collected in October 2013 from Guilin, Guangxi Province, China. All the plant materials were identified by Professor Nai-Fu Chen, Anhui Biotechnology Research Center of Plant Cell Engineering, Anhui Province, China. The voucher specimens (Table 1) are deposited at the Herbarium, College of Biotechnology and Pharmaceutical Engineering, West Anhui University, Anhui Province, China.

All the* Dendrobium* samples were dried in the sun and separately cut into small pieces, and then every 5.0 g of dried sample was investigated with the multistep analyzing procedure of polysaccharide by GC-MS and FTIR analysis.

### 2.2. Samples and Chemicals

The* Dendrobium* polysaccharides were prepared by hot-water extraction and ethanol precipitation. Briefly, the dried* Dendrobium* powders were defatted three times with 95% ethanol (w/v, 1 : 50) at 80°C, 3 h each, and then extracted three times with distilled water (w/v, 1 : 10) at 90°C, 3 h each time. The combined extracts were pooled and concentrated under a reduced pressure and then centrifuged at 4500 rpm for 15.0 min. The supernatant was collected and 100% ethanol was added slowly with stirring to precipitate the polysaccharide. The final ethanol concentration was adjusted to 76% and then kept at 4°C for 24 h. The polysaccharide pellets were obtained by centrifugation at 5000 rpm for 15 min and repeatedly washed sequentially with ethanol, acetone, and ether, respectively. The refined polysaccharide pellets were completely dissolved in appropriate volume of distilled water and repeatedly deproteinized by Sevag Method followed by filtration. Finally, the filtrate was lyophilized to yield diaphanous water-soluble* Dendrobium* polysaccharides.

Ethanol, acetone, ether, chloroform, n-butanol, methanol, hydrochloric acid, and pyridine were purchased from Sinopharm Chemical Reagent Co., Ltd. (Beijing, China). Hexamethyldisilazane and trimethylchlorosilane were bought from Aladdin Industrial Corporation (Shanghai, China). Hexane, myoinositol, xylose, arabinose, rhamnose, glucose, mannose, fructose, galactose, and galacturonic acid were bought from Sigma-Aldrich (Germany). Water was purified on a Milli-Q system (Millipore, Bedford, MA). The reagents from Sinopharm Chemical Reagent Co., Ltd., and Aladdin Industrial Corporation (Shanghai, China) are of analytical grade. The chemicals and reagents from Sigma-Aldrich are of chromatographic grade.

### 2.3. GC-MS Analysis

GC-MS analysis was performed with Trace 1300 Gas chromatograph coupled with ISQ mass spectrometer (Thermo Fisher Scientific, West Palm Beach, FL, USA) series equipment including TriPLUS RSH autosampler. The trimethylsilyl-ester O-methyl monosaccharides were separated on a TG-5 fused-silica capillary column (30 m × 0.25 mm, 0.25 *μ*m film thickness). The temperature program was 125°C to 155°C at 1.5°C/min, to 171°C at 0.8°C/min, and to 180°C at 9°C/min. The carrier gas was helium, at 1.0 mL/min, split ratio 20 : 1, and injector temperature 250°C. The injection volume was 2.0 *μ*L. The MS transfer line and ion source were at 250°C and 280°C, respectively. The MS mode was electron ionization (EI). The mass range scanned was 50–350 amu.

### 2.4. Identification and Quantification of Monosaccharides by GC-MS

The GC-MS analysis was established according to the method proposed by Guadalupe et al. [15]. The monosaccharide composition was determined by GC-MS of the trimethylsilyl-ester O-methyl glycolsyl derivants (TMS) of monosaccharides obtained after acidic methanolyisis and derivatization. 20.0 mg of polysaccharide of each* Dendrobium* sample was treated with 1.5 mL of the methanolysis reagent (MeOH containing HCl 0.5 M) in order to hydrolyse them to their corresponding methyl monosaccharides. The reaction was conducted in nitrogen atmosphere at 80°C for 16 h and thereafter the excess of reagent was removed using a stream of nitrogen gas. The conversion of the methyl glycosides to their trimethylsilyl (TMS) derivatives was performed by adding 0.5 mL of a mix of pyridine : hexamethyldisilazane : trimethylchlorosilane (10 : 2 : 1 v/v) to the dried material. The reaction was carried out at 80°C for 30 min and the reagent removed using a stream of nitrogen gas. A solution (25 *μ*L) of derivatized myoinositol was then added as internal standard and the derivative residues were extracted with 1 mL of hexane. GC-MS was performed with 2.0 *μ*L of these solutions and samples were injected in duplicate. Different standard carbohydrates were also converted to their corresponding TMS derivatives and analyzed by GC-MS in order to obtain patterns for identification and the standard calibration curves.

### 2.5. FTIR Analysis

The FTIR analysis was performed as what we had reported in the previous paper [16, 17]. FTIR spectra were recorded in the region of 4000–400 cm^−1^ on a Nicolet iS10 Fourier Transform Infrared Spectrum (Thermo Fisher Scientific, USA), equipped with a High-Performance Deuterated Triglycine Sulfate (DTGS) detector. The FTIR spectrometer was situated in an air-conditioned room (25°C, 50% relative humidity).

The freeze-dried polysaccharides from the dendrobiums were separately powdered in a blender and screened using a 200-mesh sieve. Each sample was mixed uniformly with spectroscopic grade potassium bromide (KBr) powder (1 : 100, w/w) and then pressed into a pellet before infrared spectroscopic analysis. Each spectrum was calculated from a total of 36 coadded scans with a resolution of 4 cm^−1^, and pure KBr background spectrum was recorded before analysis of the samples. Each sample was scanned with five replicates. The scans of each sample were examined for consistence and the average spectrum of each sample was used for further analyses. Prior to data analysis, each of the averaged spectra was baseline corrected and smoothed with their absorbance normalized using the Spectrum for Windows software (Nicolet OMNIC, Thermo Fisher Scientific), so that the peak absorbance of the most intense band was set to unity.

### 2.6. Similarity Evaluation

The similarities of the polysaccharides based on the GC-MS data were evaluated by the correlation coefficient (*r*_cor_) calculated by included cosine angle and using SPSS software Version 17.0 (CAMO Software AS, USA); the contents of the monosaccharide in the polysaccharides were selected as a measurement. The similarity evaluation based on the FTIR spectra was obtained using the algorithm of similarity coefficients which were automatically calculated by the software equipped in the Nicolet iS10 Fourier Transform Infrared Spectrum (Nicolet OMNIC, Thermo Fisher Scientific) [16, 17].

## 3. Results and Discussion

### 3.1. Identification and Quantification of Sugar Residues by GC-MS Detection

**Figure 1:**
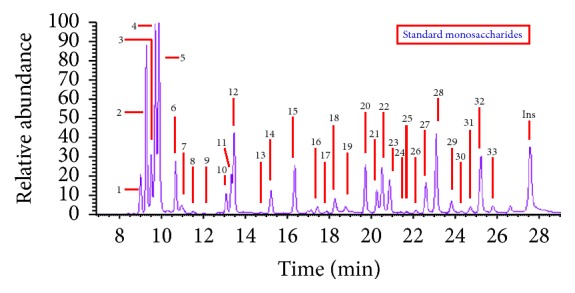
The TIC chromatograph of standard monosaccharide by TMS methyl method. Arabinose (peaks 1, 2, 5, 6, and 8), rhamnose (peaks 3, 4, 7, and 9), xylose (peaks 10–13), mannose (peaks 14–16 and 19), galactose (peaks 17, 18, 20, and 21), glucose (peaks 22, 28, 29, and 32), galacturonic acid (peaks 23–26, 30, and 31), and fucose (peaks 27 and 33).

**Figure 2:**
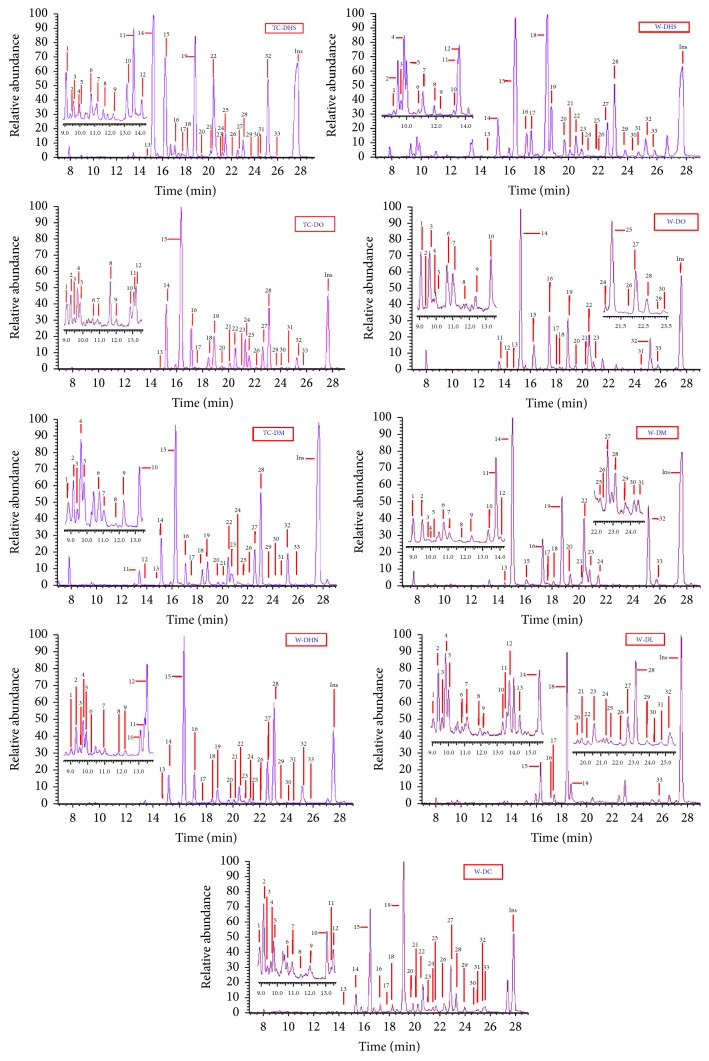
GC-MS comparison of the monosaccharides in the polysaccharides from the nine* Dendrobium* samples by TMS methyl method. Arabinose (peaks 1, 2, 5, 6, and 8), rhamnose (peaks 3, 4, 7, and 9), xylose (peaks 10–13), mannose (peaks 14–16 and 19), galactose (peaks 17, 18, 20, and 21), glucose (peaks 22, 28, 29, and 32), galacturonic acid (peaks 23–26, 30, and 31), and fucose (peaks 27 and 33).

The monosaccharide compositions of the polysaccharides in the 9* Dendrobium* samples were determined by GC-MS of their trimethylsilyl (TMS) derivatives and inositol was used as internal standard. The identification of the peaks was carried out by comparing retention times and mass spectra with those obtained by injections of pure standards. In our experiment, all the monosugars were converted to their corresponding TMS methyl glycoside derivatives and analyzed by GC-MS. Between four and seven peaks are obtained for each monosaccharide. The multiple peaks correspond to the TMS methyl glycoside derivatives of *α*- and *β*-anomers and the pyran-ring and furan-ring forms of the monosugars. Typical GC-MS chromatograms for the standard monosaccharides and the polysaccharides of the nine* Dendrobium* samples were shown in Figures 1 and 2. A total of eight kinds of monosugars including L-arabinose, rhamnose, D-xylose, D-galactose, D-glucose, D-mannose, D-galacturonic acid, and fructose were identified in each of the nine investigated dendrobiums.

In order to quantify the monosaccharides in the GC-MS chromatograms, calibration curves of the standard eight identified monosugars were detected by the ion monitoring (SIM) mode, selecting the appropriate number of ions for each compound (*m/z*) in one segment from 7 to 32 min: D-galacturonic acid, L-rhamnose, fucose, D-galactose, D-glucose, D-mannose, and D-xylose with 204 ions and L-arabinose with 217 ions. For all the spectra, these ions were their base peaks and obtained the most ion abundance and were selected for recording SIM mode chromatograms.

The features of the GC-MS detection are listed in Table 2, which includes the equation, the slope with its standard deviation, the correlation coefficients (*r* ^2^), the linear range, and the limit of detection (LOD) and the limit of quantification (LOQ) for the carbohydrate standards. The detection limits of each monosaccharide were obtained by injecting a standard mixture of derivatives as mentioned above in the derivation procedure, followed by the comparison of peak height with a signal-to-noise ratio (S/N) of three assigned limits of detection (LOD) and a signal to noise of ten assigned limits of quantification (LOQ). The features of the GC-MS method were established after a linearity study using solutions of standard carbohydrates. The analyte-to-internal-standard-peak-area ratio was used as analytical signal for constructing the calibration graphs. The limit of detection was calculated as the concentration of a signal to noise of three and the limit of quantification from the signal to noise of ten.

The correlation coefficients obtained from the calibration curves were all higher than 0.96 (*p* < 0.001). These curves were, therefore, considered to be linear for the range of concentrations studied (to 1500–1600 *μ*g for arabinose, rhamnose, mannose galactose, and glucose, 550–600 *μ*g for fucose and xylose, and 1300 *μ*g for galacturonic acid). The limits of detection and quantification were good and in all the cases they were below the values obtained for the monosaccharides present in the nine* Dendrobium* samples. The validation of the proposed method was carried out by analyzing* Dendrobium* samples. The precision of the GC-MS method was checked in terms of repeatability and reproducibility (Table 2) by means of an analysis of variance (ANOVA). Repeatability was evaluated by the analysis of six replicates of the same* Dendrobium* under normal operating conditions and it was expressed as relative standard deviation values. The reproducibility of the method was evaluated by analysis of three batches of the investigated dendrobiums and was expressed as the relative standard deviation values. As described in the Experimental, samples were defatted, extracted by hot water, precipitated by ethanol, and deproteinized by Sevag Method. The collected residues were freeze-dried, methylated, derivatizated, and submitted to GC-MS analysis to calculate the amount of carbohydrates. Both repeatability and reproducibility values were good taking into account the fact that a multistep procedure was performed with values ranging between 1 and 11% and 7 and 12%, respectively. The results of the analysis on the monosaccharide contents in the polysaccharides from the nine investigated dendrobiums were shown in Table 3, respectively.

All the investigated dendrobiums were detected to obtain the same monosaccharide compositions in our experimental conditions while the sugar contents differed remarkably among different origins and species. For example, the contents of mannose and glucose in the polysaccharide from TC-DHS were 62.0 ± 1.2 and 27.0 ± 1.1%, nearly twice those from W-DHS (32.2 ± 1.1 and 14.6 ± 0.9%, resp.) and about three times as much as those from W-DL (17.9 ± 0.9% and 11.3 ± 0.3%, resp.) (Table 3). Total 9.6 ± 0.5% of galacturonic acid was detected in the polysaccharide from TC-DO while only 0.1 ± 0.0% of galacturonic acid was checked out in W-DO, and the content of galacturonic acid in the polysaccharides from TC-DM was more than twice as much as that in its wild correspondence. The contents of galactose in W-DHS and W-DO were 25.8 ± 1.3% and 13.8 ± 0.7%, respectively, obviously higher than those in TC-DHS (2.2 ± 0.9%) and TC-DO (3.3 ± 0.3%). Thus, the nine* Dendrobium* samples might be discriminated approximately.

It was noted that a high content of mannose was detected in the polysaccharides from the investigated dendrobiums (with the mannose ratios 17.0–67.9%, Table 3). The contents of glucose, galactose, and fucose were also very high and the total content of the three monosugars in the polysaccharides was 27.5% (TC-DO, Table 3) to 71.8% (W-DL, Table 3). These results indicated that the* Dendrobium* polysaccharides were heteropolysaccharide including mannose, glucose, galactose, and fucose as major components and arabinose, xylose, rhamnose, and galacturonic acid as minor components.

The above analysis indicated that the structures of the polysaccharides between the investigated tissue-cultured dendrobiums and their wild correspondences might be different. Furthermore, the variations of the contents of monosaccharides might provide new approaches in the discrimination and identification of the nine dendrobiums.

### 3.2. Similarity Analysis of GC-MS Fingerprint

To evaluate the similarity of chemical compositions of different origins and species of dendrobiums directly, the correlation coefficients (*r*_cor_) were calculated by cosine angle method based on the data of monosugars in polysaccharides and were used as similarity measure. As it was shown in Table 4, *r*_cor_ values between W- and TC-DHS, W- and TC-DO, and W- and TC-DM were only 0.831, 0.865, and 0.884, respectively, and *r*_cor_ values ranged from 0.475 to 0.837 across species. In the term of the compositions and the contents of the monosaccharides, the similarities between different origins of* Dendrobium* samples were higher than those across species, and the differences of the polysaccharide in tissue-cultured dendrobiums and their wild correspondences might be more than what we had proposed though they were cultivated in the same conditions.

### 3.3. FTIR Spectroscopy Analysis of Polysaccharides

**Figure 3:**
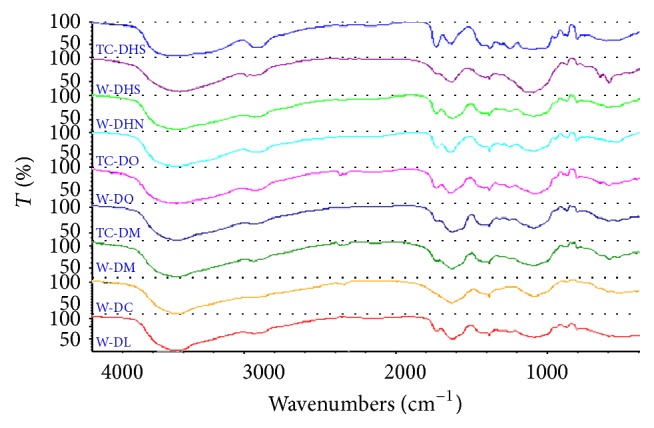
The FTIR spectra of the polysaccharides of seven* Dendrobium* samples in the ranges of 4000–400 cm^−1^.

Fourier transform infrared spectroscopy (FTIR) is an important method to predict the structures of natural macromolecules such as polysaccharide and is an alternative method for TCM discrimination respecting the notion that the macrofingerprint features of FTIR spectra are consistent with the holistic theory of TCM. Considering the notion that the similarity analysis based on the monosugar contents might not effectively reflect the overall structural variations among the polysaccharides from different origins and species, the FTIR spectrum was investigated to further evaluate the similarity among the nine* Dendrobium* samples.  Figure 3 showed the IR spectra of the nine* Dendrobium* polysaccharides, respectively. All samples' IR spectrum had a broad stretching intense characteristic peak at around 3425 ± 4 cm^−1^ for OH and a weak peak at around 2935 ± 4 cm^−1^ for CH bond. The peaks at around 1650 ± 4 cm^−1^ and 1380 ± 4 cm^−1^ were attributed to the bending vibration of C=O. The characteristic peak around 860 ± 4 cm^−1^ implied the presence of *β*-D-glycosidic linkages in the polysaccharides.

### 3.4. Similarity Analysis of FTIR Fingerprint

The similarity analysis based on the IR spectra of the polysaccharides from the 9* Dendrobium* samples was shown in Table 5. The similarities between different origins of DHS, DO, and DM were only 86.8–88.7% though they were higher than those between species of the investigated dendrobiums, consistent with the results of GC-MS analysis, further confirming that the differences of the polysaccharides structure might exist between tissue-cultured dendrobiums and their wild correspondences.

## 4. Conclusion

In conclusion, the results obtained in this study can provide comprehensive evaluation for quality between tissue-cultured and wild dendrobiums and offer an optimized evaluation method for medicinal herb quality control. A total of 8 monosugars with different contents were identified in the polysaccharide from each of the nine* Dendrobium* samples. The similarity evaluation based on GC-MS data showed that *r*_cor_ values of different origins of* D. huoshanense*,* D. officinale*, and* D. moniliforme* were 0.831, 0.865, and 0.884, respectively, while *r*_cor_ values ranged from 0.475 to 0.837 across species. The similarity evaluation based on FTIR files of the polysaccharides showed that the similarity coefficients between W and TC* D. huoshanense*,* D. officinale*, and* D. moniliforme* were 88.7%, 86.8%, and 88.5%, respectively, in contrast to the similarity coefficients varying from 57.4% to 82.6% across species. The results revealed that although the genetic information was the same between tissue-cultured dendrobiums and their wild correspondences and was very similar among the different* Dendrobium *spp., the monosaccharide compositions as well as the whole structure of the polysaccharides might be quite different between origins and across species. How to keep the similarity of the main bioactive components including polysaccharide between the tissue-cultured medicinal plants and their wild correspondences would be a great challenge and bottleneck in the conversation and utilization of the in-danger* Dendrobium *species such as* D. huoshanense* using tissue-culture technology.

## Figures and Tables

**Table 1:** The list of *Dendrobium *samples.

Samples	Abbreviation	Location	Gatherer	Voucher number
Wild *D. huoshanense*	W-DHS	Huoshan, Anhui, China	Jun Dai	201310HS0101Y
Tissue-cultured *D. huoshanense*	TC-DHS	Huoshan, Anhui, China	Jun Dai	201310HS0101T
Wild *D. officinale*	W-DO	Huoshan, Anhui, China	Jun Dai	201310HS0201Y
Tissue-cultured *D. officinale*	TC-DO	Huoshan, Anhui, China	Jun Dai	201310HS0201T
Wild *D. moniliforme*	W-DM	Huoshan, Anhui, China	Jun Dai	201310HS0301Y
Tissue-cultured *D. moniliforme*	TC-DM	Huoshan, Anhui, China	Jun Dai	201310HS0301T
Wild *D. henanense*	W-DHN	Huoshan, Anhui, China	Jun Dai	201310HS0401Y
Wild *D. loddigesii*	W-DL	Guilin, Guangxi, China	Jun Dai	201310GX0501Y
Wild *D. crepidatum*	W-DC	Guilin, Guangxi, China	Jun Dai	201310GX0501Y

**Table 2:** Calibration curve data, calculated detection (LOD), and quantification (LOQ) limits and precision (repeatability and reproducibility) of the monosaccharide standards detected by GC-MS.

Compounds	Equation	Sd_slope_	Correlation coefficient (*r* ^2^)	Linear range (*μ*g)	LOD (*μ*g)	LOQ (*μ*g)	Reproducibility (%)	Repeatability (%)
Arabinose	*A* = 0.607*C*	0.008	0.985	0.5–1600	0.2	0.8	12	6
Rhamnose	*A* = 0.652*C*	0.090	0.975	0.5–1600	0.2	0.5	9	11
Xylose	*A* = 0.547*C*	0.007	0.989	0.5–600	0.1	0.5	7	6
Mannose	*A* = 0.811*C*	0.092	0.993	2.5–1500	0.1	0.6	7	3
Galactose	*A* = 0.392*C*	0.010	0.987	1.5–1500	0.3	0.7	7	5
Glucose	*A* = 0.913*C*	0.023	0.998	1.0–1600	0.1	1.8	7	5
Galacturonic acid	*A* = 0.238*C*	0.005	0.977	4.0–1300	1.0	5.0	9	7
Fucose	*A* = 0.566*C*	0.010	0.969	0.5–550	0.4	1.2	12	9
Inositol	*A* = 0.954*C*	0.035	0.993	1.0–1500	0.2	1.5	10	1

**Table 3:** Monosaccharide compositions and contents (%) of the polysaccharides from the nine *Dendrobium *samples detected by GC-MS analysis of their TMS derivatives.

Com.	TC-DHS	W-DHS	TC-DO	W-DO	TC-DM	W-DM	W-DHN	W-DL	W-DC
Arabinose	0.6 ± 0.1	2.8 ± 0.4	0.1 ± 0.0	0.2 ± 0.1	0.7 ± 0.1	0.6 ± 0.1	0.4 ± 0.1	1.2 ± 0.1	0.5 ± 0.1
Rhamnose	0.3 ± 0.1	2.7 ± 0.3	0.1 ± 0.0	0.2 ± 0.0	0.7 ± 0.1	0.5 ± 0.1	0.4 ± 0.1	1.1 ± 0.1	0.6 ± 0.1
Xylose	0.3 ± 0.1	3.0 ± 0.2	0.1 ± 0.1	0.2 ± 0.1	0.3 ± 0.1	0.2 ± 0.1	1.0 ± 0.1	0.9 ± 0.2	0.5 ± 0.1
Mannose	62.0 ± 1.2	32.2 ± 1.1	65.6 ± 2.3	59.5 ± 1.5	48.5 ± 1.3	60.0 ± 1.7	50.6 ± 1.4	17.9 ± 0.9	59.7 ± 2.1
Galactose	2.2 ± 0.9	25.8 ± 1.3	3.3 ± 0.3	13.8 ± 0.7	3.9 ± 0.1	2.3 ± 0.2	2.5 ± 0.2	41.8 ± 1.7	3.8 ± 0.2
Glucose	27.0 ± 1.1	14.6 ± 0.9	17.9 ± 1.1	18.4 ± 1.2	29.8 ± 0.9	30.9 ± 1.3	29.9 ± 1.3	11.3 ± 0.3	10.4 ± 0.6
Galacturonic acid	4.1 ± 0.8	3.3 ± 0.2	9.6 ± 0.5	0.1 ± 0.0	5.1 ± 0.5	2.3 ± 0.1	2.6 ± 0.2	4.8 ± 0.3	6.5 ± 0.3
Fucose	1.2 ± 0.1	11.9 ± 0.7	4.5 ± 0.4	4.1 ± 0.4	10.2 ± 0.8	1.8 ± 0.1	10.1 ± 0.5	16.7 ± 1.1	16.7 ± 0.8

Values are means ± standard deviations (*n* = 3).

**Table 4:** The correlation coefficients of the nine *Dendrobium* samples.

	TC-DHS	W-DHS	TC-DO	W-DO	TC-DM	W-DM	W-DHN	W-DL	W-DC
TC-DHS	1	0.831	0.780	0.764	0.775	0.795	0.777	0.475	0.719
W-DHS		1	0.738	0.764	0.736	0.726	0.732	0.787	0.750
TC-DO			1	0.865	0.837	0.760	0.738	0.494	0.765
W-DO				1	0.729	0.754	0.731	0.683	0.735
TC-DM					1	0.884	0.797	0.521	0.795
W-DM						1	0.784	0.479	0.794
W-DHN							1	0.502	0.701
W-DL								1	0.243
W-DC									1

**Table 5:** The similarity coefficients of the FTIR of the polysaccharides from the nine dendrobiumsin the ranges of 4000–400 cm^−1^ (%).

Similarity coefficients	TC-DHS	W-DHS	TC-DO	W-DO	TC-DM	W-DM	W-DHN	W-DL	W-DC
TC-DHS	100	88.7	77.9	75.7	75.0	79.4	77.5	61.1	70.1
W-DHS		100	62.2	67.9	75.6	82.3	74.9	68.1	67.1
TC-DO			100	86.8	83.7	77.9	80.1	58.6	81.9
W-DO				100	80.1	77.8	76.1	71.2	78.4
TC-DM					100	88.5	82.6	63.4	73.1
W-DM						100	80.5	68.8	76.2
W-DHN							100	57.4	78.5
W-DL								100	77.4
W-DC									100
